# Attractiveness of host banana leaf materials to the banana weevil, Cosmopolites sordidus in Ghana for development of field management strategies

**DOI:** 10.1002/ps.5182

**Published:** 2018-10-22

**Authors:** Samson A Abagale, Christine M Woodcock, Keith Chamberlain, Samuel Osafo‐Acquaah, Helmut van Emden, Michael A Birkett, John A Pickett, Haruna Braimah

**Affiliations:** ^1^ Crops Research Institute, Council for Scientific and Industrial Research Kumasi Ghana; ^2^ Department of Chemistry Kwame Nkrumah University of Science and Technology Kumasi Ghana; ^3^ Biointeractions and Crop Protection Department Rothamsted Research Harpenden UK; ^4^ School of Agriculture, Policy and Development The University of Reading Reading UK; ^5^ School of Chemistry Cardiff University Cardiff UK

**Keywords:** banana leaves, olfactometer, attraction, banana weevil, palm wine alcohol, electrophysiology

## Abstract

**BACKGROUND:**

The banana weevil, *Cosmopolites sordidus*, has been frequently cited as the most challenging constraint to banana and plantain production, particularly in small‐scale (smallholder) farming. For the development of a new, low‐cost weevil management technology based on attractive host plant material, we previously identified (2*R*,5*S*)‐theaspirane as the active component of attractive senesced banana leaves. In this new study, we used behavioural (olfactometer) bioassays with adult weevils to compare the attractiveness of four different developmental stages of banana leaves, i.e. unfolding (pale green), matured green (deep green), matured yellowing and senesced, to determine which leaf developmental stage would be most appropriate for use in weevil management. We also investigated the attractiveness of senesced leaf extracts prepared using different solvents to determine which solvent would be most appropriate for local production of leaf extracts. Coupled gas chromatography–electroantennography (GC–EAG) was then used with adult weevils to confirm the presence of (2*R*,5*S*)‐theaspirane in attractive leaf extracts.

**RESULTS:**

Of the leaf materials tested, only the odour of senesced leaf material was significantly attractive to adult weevils (*P* < 0.005). Furthermore, an extract of senesced material prepared using palm wine alcohol was significantly attractive (*P* < 0.05). Using coupled GC–EAG with weevil antennae, (2*R*,5*S*)‐theaspirane was identified as a minor component with strong EAG activity within the palm wine alcohol extract.

**CONCLUSION:**

The results suggest that palm wine alcohol extracts of senesced banana leaf material could be used to lure adult *C. sordidus* to traps in the field, as part of an ethnobotanical‐based approach for *C. sordidus* management on smallholder farms. © 2018 The Authors. *Pest Management Science* published by John Wiley & Sons Ltd on behalf of Society of Chemical Industry.

## 1 INTRODUCTION

Bananas and plantains, *Musa* spp., are the fourth most important crop in humid tropics, with worldwide banana production estimated at > 100 Mt in 2015.^1^ The banana weevil, *Cosmopolites sordidus* Germar (Coleoptera, Curculionidae), is reported to be the most challenging constraint to banana and plantain production, particularly in small‐scale (smallholder) farming.^2^, ^3^, ^4^ Adult *C. sordidus* are free‐living, nocturnal, long‐lived and breed in banana debris or fallen mats,^5^ and although they move freely within banana stands, few disperse more than 50 m in 3 months.^6^ Weevils forage for food by detecting volatiles emanating from the host plant,^7^, ^8^, ^9^, ^10^, ^11^, ^12^ and males produce an aggregation pheromone^13^ to which both sexes respond.^14^, ^15^ Females deposit eggs at the base of the pseudostem or on exposed corms, and the larvae develop into pupae within 15–20 days after passing through five to eight instars. When the larvae emerge, they tunnel through the corm to feed and develop. The feeding and tunnelling behaviour damages the corm and weakens the plant. This reduces water and mineral uptake, resulting in reduction of bunch weight (yield) and causing plant toppling during windstorms.^16^ In severe weevil infestations, crop losses of up to 100% have been reported.^17^

Numerous approaches for *C. sordidus* management have been explored, including habitat manipulation through cultural control systems,^12^, ^18^, ^19^ biological control, use of botanical and conventional pesticides,^12^ and combinations of these approaches.^19^ However, the ecology of adult *C. sordidus*, and the tunnelling feeding behaviour of the immature stages, make it difficult to monitor and control populations by conventional methods.^20^ Because *C. sordidus* can crawl and is not prone to dispersal, mass trapping using semiochemicals (naturally occurring behaviour‐modifying chemicals) is a promising approach for control,^21^ with trapping systems also being a potential means of luring weevils to encounter killing agents, i.e. pesticides or other killing agents such as entomopathogens.^8^, ^22^ The male‐produced aggregation pheromone of *C. sordidus* has been identified as (1*S*,3*R*,5*R*,7*S*)‐sordidin and commercialized for weevil trapping,^13^, ^23^, ^24^, ^25^, ^26^, ^27^ but although the aggregation pheromone has been produced on a large scale for field tests^24^, ^25^ and commercialized,^26^ the technology is expensive for smallholder farmers.

The attractiveness of host plant volatiles (kairomones) to various weevil species has been reported,^10^, ^28^, ^29^, ^30^, ^31^, ^32^ with kairomones being used to either enhance trapping systems, aggregate populations for the deployment of other pest management interventions,^33^ or enhance the attractiveness of pheromone‐baited traps.^14^, ^34^, ^35^ In our earlier studies on the chemical ecology of *C. sordidus*, we observed that adult weevils were highly attracted to senesced (naturally dried) banana leaf material, and identified the active component using behavioural (olfactometer) bioassays and coupled gas chromatography–electroantennography (GC–EAG) as (2*R*,5*S*)‐theaspirane.^9^, ^10^, ^11^ From a smallholder farmer perspective, host plant‐derived attractants that can be produced locally from readily available and renewable materials are much more affordable and sustainable than commercially produced synthetic insect pheromones. Here, we investigate the responses of adult *C. sordidus* to odours of senesced banana leaf material in comparison with three other different developmental stages of host banana leaf material, i.e. unfolding (pale green) banana leaf, matured green (deep green) banana leaf and matured yellowing banana leaf, to identify which stage produces odours that are most attractive to *C. sordidus*. We also studied the behavioural activity of extracts of senesced leaf material prepared using different solvents, i.e. methanol, ethanol, hexane and palm wine alcohol, to determine which would be most suitable for generating attractive leaf extracts, and used coupled GC–EAG to confirm the presence of the previously identified (2*R*,5*S*)‐theaspirane in attractive solvent extracts. Identification of the most attractive leaf developmental stage would underpin the development of new, low‐cost weevil management technology that is affordable for smallholder banana/plantain farmers, and determination of energy requirements for leaf extraction would enable us to assess feasibility for the scaling up and local production of the *C. sordidus* attractant.

## 2 MATERIALS AND METHODS

### 2.1 Banana leaves

Banana leaf material required for experiments was collected from banana plants on smallholder farms near Kwadaso, Kumasi, Ghana. Freshly collected material was collected at three different stages of leaf development, i.e. unfolding (pale green) banana leaf, matured green (deep green) banana leaf and matured yellowing banana leaf. Portions of the freshly collected materials were air dried in the laboratory under shade for 10 days. Completely senesced (naturally dried) leaf material was also collected from smallholder farms. Samples of the freshly collected leaf material, freshly collected and dried leaf material, and senesced banana leaf material were used in subsequent experiments.

### 2.2 Weevil culture

Adult *C. sordidus* were collected from banana fields around the CSIR‐CRI Experimental Station at Kwadaso in Kumasi, Ghana and brought to the Chemical Ecology Laboratories at CSIR‐CRI and Rothamsted Research, UK. The weevils were cultured in plastic containers as follows.^10^ Each container was provided with pieces of banana rhizomes as food. The bottom of each container was lined with tissue paper moistened with distilled water to provide a moist environment for the weevils. The weevil culture was kept at room temperature and inspected daily to remove dead weevils; the tissue mats were replaced or moistened weekly. The weevils and containers were also cleaned using running tap water, and the feed rhizomes replaced every month.^36^ Weevils used in the experiments were adult unsexed members taken from within the weevil culture, placed in a container without food (rhizome) and starved for at least 12 h before use.

### 2.3 Volatile collection

Volatile organic compounds (VOCs) from senesced leaf material were collected by air entrainment (dynamic headspace collection) using standard techniques.^37^ Air filtered through activated charcoal was pumped into a glass jar (5 L) containing 80–100 g of senesced leaf material at a rate of 900 mL min^−1^ and the VOCs trapped on Porapak Q (50 mg, 50–80 mesh) packed in a glass tube using silanized glass wool (Supelco) at both ends. Trapped VOCs collected over 72 h were eluted with distilled diethyl ether (750 µL). The resultant eluants were stored at −20 °C in tightly capped microvials until required for behavioural and electrophysiological studies.

### 2.4 Solvent extraction

Solvent extracts of senesced banana leaves required for behavioural and electrophysiological experiments were prepared in the organic chemistry laboratory of Kwame Nkrumah University of Science and Technology (KNUST), Ghana. Extracts using ethanol, methanol or hexane (1:3 w/v) were prepared by either cold maceration or Soxhlet extraction. A cold maceration extract using palm wine alcohol as the solvent (1:2 w/v) was also prepared. For cold maceration, leaves were left to soak in solvent in glass jars for 24 h at ambient temperature, with periodic shaking. For Soxhlet extraction, extraction of leaf material in refluxing solvent was carried out using a heating mantle (temperature set to 70 °C) until the redistilled solvent became colourless. Solvent extracts from cold maceration and Soxhlet extraction were decanted into Erlenmeyer flasks, concentrated under a gentle stream of nitrogen to a volume of ∼ 15 mL and stored in tightly capped vials at −4 °C until required for behaviour and electrophysiology, whereupon they were further concentrated to a volume of 1 mL.

### 2.5 Olfactometry

A linear three‐chambered olfactometer comprising three identical round Perspex chambers, as described previously,^38^ was used to assess the behavioural response of adult *C. sordidus* to plant materials, collected VOCs and solvent extracts. The middle chamber was the test or release chamber, whereas the two side chambers acted as the response chambers (one chamber for the control stimulus and the other for the test stimulus).^11^, ^39^ Each of the three chambers was ∼ 900 mm internal diameter and ∼ 500 mm high, and the chambers were linked to each other by narrow tubes (100 mm internal diameter, also made of Perspex) to allow movement of weevils from one chamber to another. Black tape was used to cover the entire outer surface of the set‐up to make it dark and opaque. Air vents were created on the left and right ends of the two response chambers, and these vents were connected to charcoal filters through which filtered air entered the chambers. Behavioural experiments were conducted to evaluate the attractiveness of fresh and laboratory‐dried banana leaves, and senesced banana leaves. Choice tests were done to compare the attractiveness of dried and senesced banana leaves, and compare the attractiveness of senesced banana leaves with their VOCs. In tests with leaf material, the test sample was placed in one of the test chambers of the olfactometer and the second test chamber was left empty to serve as a blank control. In choice tests, the same mass of leaf material was placed in each of the test chambers. In tests using VOC samples, 20 µL of VOC extract (equivalent to 0.08 g leaf material) was applied on equally sized 1 cm^2^ pieces of Whatman® qualitative filter paper, Grade 1 (Sigma‐Aldrich, Gillingham, UK) in each test, and the solvent allowed to evaporate (∼ 10 s) before use. Filter papers with solvent were used as controls. In tests using solvent extracts, 20 µL equivalent quantities of extract and pure solvent were applied on the filter paper. The solvents were allowed to evaporate (∼ 10 s) before the pieces of paper were placed in the olfactometer. For each assay, groups of 10 unsexed, adult weevils removed from the weevil culture were used.^36^ Test weevils were placed in the main chamber, and allowed 20–30 min to respond to and move towards stimuli in either response chamber. After the first test period, if two (20%) or more weevils failed to respond to either test material, a further 10–15 min was allowed for them to respond. At the end of the permitted time, the numbers of weevils in each chamber were recorded. For each experiment, a total of 200 different weevils were used in 20 replicates. The test materials were switched between chambers midway through the experiment (after the first 10 replicates). Before the switch of test materials, the apparatus was cleaned with dilute ethanol, thoroughly rinsed in distilled water, wiped dry with tissue paper and allowed to air dry for 30 min.

### 2.6 Data analysis

Olfactometry data were analysed using proportionate analyses based on the assumption that ordinarily randomly moving weevils would respond to test materials equally. *T*‐tests at a significant of *P* < 0.05 were used to determine the differences in attractiveness between the test materials. Non‐responders were removed from the analyses.

### 2.7 Electrophysiology

Electroantennogram recordings from the antenna of unsexed adult *C. sordidus* were made using Ag–AgCl glass electrodes filled with saline solution composed as described elsewhere,^40^ but without glucose. An antenna was excised and suspended between the two electrodes. The tip of the terminal process of the antenna was removed to ensure a good contact. The signals were passed through a high‐impedance amplifier (UN‐06, Syntech, Hilversum, The Netherlands) and analysed using a customized software package (Syntech).

### 2.8 Coupled gas chromatography–electroantennography

The coupled GC–electrophysiology system, in which the effluent from the GC column is simultaneously directed to the antennal preparation and the GC detector, has been described previously.^41^ Separation of the collected palm wine alcohol extract was achieved by high‐resolution GC (6890N, Agilent Technologies, Stockport, UK) equipped with a cool on‐column injector and flame ionization detector (FID). A capillary GC column was used, 50 m × 0.32 mm i.d. DB‐1 column (Agilent J & W, Agilent Technologies, Stockport, UK). The oven temperature was maintained at 30 °C for 2 min and then programmed to increase at 10° min^−1^ to 250 °C. The carrier gas was helium. The outputs from the EAG amplifier and the FID were monitored simultaneously and analysed using the software package (Syntech). A peak was deemed to be electrophysiologically active if it elicited responses on three or more antennal preparations.

### 2.9 Coupled gas chromatography–mass spectrometry

Coupled gas chromatography–mass spectrometry (GC‐MS) analysis was performed using a Waters Autospec Ultima mass spectrometer (+EI, 70 eV, source temperature 250 °C, *m*/*z* 40–500) coupled to an Agilent 6890 GC fitted with a DB‐1 capillary column (J & W Scientific, 50 m × 0.32 mm id × 0.52 µm film thickness) and a cool on‐column injector. The oven temperature was programmed to start at 30 °C for 5 min, then increase at 5 °C min^−1^ until 250 °C, with a final hold of 10 min. The carrier gas was helium. Tentative identification of the EAG‐active compound for *C. sordidus* was confirmed by comparison of GC retention time and peak enhancement using an authentic sample of (2*R*,5*S*)‐theaspirane.^11^

## 3 RESULTS

**Figure 1:**
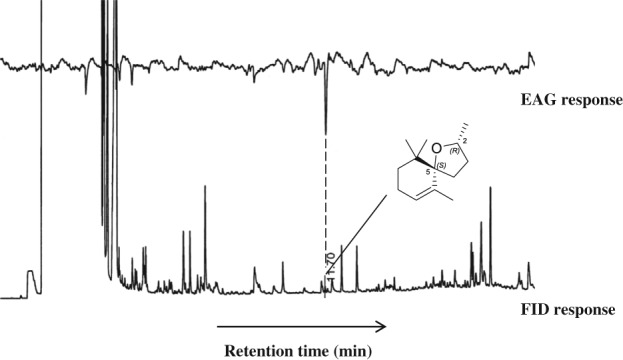
Coupled gas chromatography‐electroantennography (GC‐EAG) responses of adult Cosmopolites sordidus to senesced banana leaf palm wine alcohol extract on a non‐polar DB‐1 GC column. Lower trace = FID response. Upper trace = EAG response. Annotated peak identified by coupled GC‐MS analysis and GC peak enhancement as (2*R*,5*S*)‐theaspirane (structure included).

In olfactometer assays, both freshly collected and freshly collected, laboratory‐dried samples of banana leaf material, from different developmental stages, i.e. unfolding (pale green) banana leaf, matured green (deep green) banana leaf and matured yellowing banana leaf, were not statistically more attractive to adult *C. sordidus* than the clean air control (Table 1). Senesced banana leaf material, however, was significantly more attractive to weevils than the clean air control (*P* < 0.005, Table 1). In choice assays, the senesced leaf material was more attractive than the dried unfolding banana leaf sample (*P* < 0.03, Table 1). An extract of VOCs collected from senesced leaf material by air entrainment was significantly more attractive to adult weevils than a solvent control (*P* < 0.001, Table 1). In choice assays, there was no significant difference between the attractiveness of senesced leaf material and collected VOCs (Table 1) to banana weevils. A solvent extract of senesced leaves prepared using palm wine alcohol was significantly more attractive when compared with a solvent control, whereas extracts prepared using methanol, ethanol and hexane did not differ from solvent control in attractiveness to the test weevils (Table 2). There were no statistical differences in attractiveness of extracts made at ambient temperatures and those extracted using Soxhlet apparatus (Table 2). When compared with whole leaf material, only the methanolic extract was significantly attractive (Table 2). In olfactometry assays to ascertain the comparative attractiveness of extracts of senesced leaves in the various solvents to *C. sordidus*, palm wine alcohol extract was similar to those made in methanol and hexane but was significantly more attractive than the ethanol extract (Table 2). Coupled GC–electroantennography (GC–EAG) analysis, on a non‐polar DB‐1 GC column, of the palm wine alcohol extract (the more attractive extract) using adult weevil antennae revealed the presence of a minor component with significant EAG activity (Fig. 1), which was identified by coupled GC–MS and GC peak enhancement with an authentic standard as (2*R*,5*S*)‐theaspirane.

**Table 1:** Responses of adult banana weevils, Cosmopolites sordidus, to different developmental stages of banana leaf material (freshly harvested, laboratory dried and senesced) in a linear olfactometer (*N* = 20 replicates per experiment). Response measured as the mean (± SE) number of weevils found in each chamber of the olfactometer at the end of the experiment. Data were analysed by Student's *t*‐test

Treatment A	Mean ± SE	Treatment B	Mean ± SE	*P*‐value
Fresh unfolded pale green banana leaf	5.50 ± 0.43	Clean air	4.15 ± 0.42	NS
Fresh mature green banana leaf	5.40 ± 0.44	Clean air	4.40 ± 0.43	NS
Fresh mature yellowing leaf	4.70 ± 0.50	Clean air	5.15 ± 0.41	NS
Dried unfolded pale green banana leaf	4.60 ± 0.43	Clean air	5.05 ± 0.48	NS
Dried mature green banana leaf	5.35 ± 0.53	Clean air	4.35 ± 0.50	NS
Dried mature yellowing leaf	4.55 ± 0.42	Clean air	4.80 ± 0.46	NS
Senesced banana leaf	6.00 ± 0.46	Clean air	3.80 ± 0.32	< 0.005
Senesced banana leaf	5.70 ± 0.36	Dried unfolded pale green banana leaf	3.85 ± 0.36	< 0.03
Senesced banana leaf	5.00 ± 0.36	Dried mature green banana leaf	4.10 ± 0.31	NS
Senesced banana leaf	5.10 ± 0.38	Dried mature yellowing leaf	4.65 ± 0.37	NS
Senesced banana leaf volatile organic compounds	6.30 ± 0.41	Diethyl ether	3.20 ± 0.42	0.001
Senesced banana leaf volatile organic compounds	4.90 ± 0.51	Senesced banana leaf	4.45 ± 0.51	NS

NS, no significant difference between treatments

**Table 2:** Responses of adult banana weevils, Cosmopolites sordidus, to senesced banana leaf material, collected volatile organic compounds (VOCs) and solvent extracts (prepared using different solvents at ambient temperature and by Soxhlet extraction) in a linear olfactometer (*N* = 20 replicates per experiment). Response measured as the mean (± SE) number of weevils found in each chamber of the olfactometer at the end of the experiment. Data were analysed by Student's *t*‐test

Treatment A	Mean ± SE	Treatment B	Mean ± SE	*P*
Leaf material	5.60 ± 0.43	Clean air	3.45 ± 0.40	0.014
Leaf material VOCs	6.50 ± 0.39	Diethyl ether	3.05 ± 0.38	< 0.0002
Leaf material	4.65 ± 0.47	Leaf material VOCs	4.40 ± 0.51	NS
Methanol extract	5.45 ± 0.49	Methanol	3.80 ± 0.46	NS
Ethanol extract	5.00 ± 0.48	Ethanol	4.55 ± 0.47	NS
Hexane extract	4.85 ± 0.36	Hexane	4.15 ± 0.40	NS
Palm alcohol extract	5.70 ± 0.49	Palm alcohol	3.75 ± 0.38	< 0.05
Methanol extract	4.35 ± 0.40	Soxhlet methanol extract	4.55 ± 0.34	NS
Ethanol extract	5.75 ± 0.41	Soxhlet ethanol extract	3.70 ± 0.47	NS
Hexane extract	4.45 ± 0.51	Soxhlet hexane extract	4.20 ± 0.51	NS
Leaf material	5.80 ± 0.36	Methanol extract	3.95 ± 0.36	0.03
Leaf material	4.35 ± 0.48	Ethanol extract	4.30 ± 0.51	NS
Leaf material	4.90 ± 0.41	Hexane extract	4.15 ± 0.43	NS
Leaf material	4.10 ± 0.44	Palm alcohol extract	4.80 ± 0.43	NS
Palm alcohol extract	5.20 ± 0.32	Methanol extract	4.65 ± 0.33	NS
Palm alcohol extract	6.15 ± 0.54	Ethanol extract	3.45 ± 0.49	< 0.04
Palm alcohol extract	5.15 ± 0.52	Hexane extract	4.05 ± 0.52	NS

NS, no significant difference between treatments.

## 4 DISCUSSION

Olfactometry is used to investigate the role of volatile semiochemicals in insect host location independently from visual stimuli.^42^ Previous studies on the chemical ecology of adult banana weevils, *C. sordidus*, showed that senesced banana leaf material was highly attractive in olfactometer bioassays, whereas fresh plant material, including wounded tissue, was not attractive.^9^, ^10^, ^11^ Furthermore, senesced leaf material was reported to be more attractive when compared with banana rhizome and pseudostem, cocoyam and dead grasses.^9^, ^10^ In this study, banana leaves that were harvested as fresh materials and those dried artificially in the laboratory, i.e. unfolding (pale green) leaf, matured green (deep green) leaf and matured yellowing leaf material, were not significantly attractive, whereas senesced leaf material was significantly more attractive compared with clean air (Table 1). Comparing the three types of artificially dried leaf material to senesced leaf material, significant attraction to the senesced leaf material was only observed when compared with the dried unfolding pale green leaf material. There was no significant difference in attractiveness between the senesced leaves, the dried matured green leaves and the dried matured yellowing leaves. These results suggest that of the leaf stages tested, senesced banana leaf material produces volatile attractant(s). However, the relationship between leaf age and production of volatile attractant(s) remains unclear.

Insect host location frequently involves the detection and utilization of host‐derived volatile semiochemicals. Several insect species recognize suitable hosts by detecting key volatiles that are either taxonomically distinct or are ubiquitous across different taxa but present in specific ratios.^42^ In this study, attraction of adult *C. sordidus* to both senesced banana leaf material and collected VOCs confirmed our earlier work that both contain volatile attractant(s), and that leaf development through senescence might be important for production of the attractant(s) in banana leaf tissue. The attractiveness of collected VOCs was more significant (*P* < 0.001) than senesced leaf material (*P* < 0.005) when compared with controls. However, there was no significant difference in attractiveness between the two samples when they were tested in a dual‐choice olfactometer bioassay, suggesting that the previously identified (2*R*,5*S*)‐theaspirane may account for the attractiveness of senesced banana leaf material.^11^

Attractive volatile semiochemicals provide a means of selective removal of target pest species in pest management.^43^ Mass trapping schemes that utilize semiochemicals to manage weevil species have been developed.^44^, ^45^ For the banana stem weevil, *Odoiporus longicollis* Olivier, it was suggested that identification of semiochemicals can facilitate the design of simple cost‐effective traps that are easy to use by smallholder farmers.^39^ It has also been suggested that no single control strategy is likely to provide complete control for banana weevils and that an IPM strategy encompassing other components of pest management might provide the best chance for successfully managing this pest.^46^ Although it has been shown that pheromone traps are not always effective for *C. sordidus* at all places,^27^ they were more effective than pseudostem traps and were used successfully in Costa Rica, Uganda and South Africa.^47^ Mass trapping, as a pest management tool directed against adult insects, has the intention of selectively extracting a pest species and thereby suppressing its population to a level below the threshold of damage.^48^ For *C. sordidus*, split pseudostem and rhizome traps have been traditionally used,^5^, ^49^, ^50^ but additionally, other parts of the plant that are attractive to the pest have also been used.^13^ However, these plant materials are only attractive over short periods of between 7 and 14 days, depending on environmental conditions.^51^ In comparing the solvent extracts of senesced banana leaf material prepared at ambient temperature, palm wine alcohol extract was significantly more attractive to *C. sordidus* than the solvent control. Furthermore, the attractiveness of palm wine alcohol extracts of senesced banana leaf material, and the presence of the attractant (2*R*,5*S*)‐theaspirane as demonstrated by coupled GC–EAG suggests the potential for using such extracts, instead of bulky banana leaf materials, as bait material for *C. sordidus* trapping, with the extracts also being easier to preserve than corms, rhizomes and whole‐leaf material. Further work is required in application of findings in this work since attractiveness of kairomones can be influenced by several factors.^42^, ^52^

Studies conducted elsewhere have shown that pounded and chopped corms and pseudostems do not aggregate high numbers of *C. sordidus*,^53^ despite earlier reports of attraction by pseudostems and corms.^5^, ^49^, ^50^ Thus, processing of attractive plant parts may influence their effectiveness as sources of attractant compounds. In this study, palm wine alcohol extracts of senesced banana leaves were prepared at ambient temperature and shown to be attractive to *C. sordidus* in the laboratory. Further studies are needed to confirm the ability of palm wine alcohol extracts of to lure *C. sordidus* to traps in the field. Higher responses of *C. sordidus* to combinations of fermented banana tissues and aggregation pheromone in olfactometer bioassays have been reported,^54^ and combinations of host plant material and attractants such as pheromones has been suggested as a means of improving the efficacy and longevity of weevil traps in the field.^55^ The additive and/or synergistic activity of such combinations have been reported for several weevil species^56^, ^57^ and also for *C. sordidus*.^13^ Other studies reported that the combination of aggregation pheromone with other materials can be used for efficient trapping and suppression of banana weevil populations^58^ and that host plant extracts enhance the attractiveness of pheromone to *C. sordidus*.^59^, ^60^ Therefore, future work will need to also need to test combinations of palm wine alcohol extracts with the aggregation pheromone (1*S*,3*R*,5*R*,7*S*)‐sordidin for potential synergistic or additive effects. The low energy requirement for preparation of senesced banana leaf extracts suggests feasibility for the scaling up and local production of the *C. sordidus* attractant.

## 5 CONCLUSION

Senesced banana leaf material and collected VOCs were found to be significantly attractive to adult banana weevils, whereas similar attractiveness was not recorded in leaf material that was harvested fresh and artificially dried. There was no significant difference in attraction between the senesced material and the collected VOCs, implying successful isolation of the attractive component(s). Furthermore, a palm wine alcohol extract of senesced banana leaf material was attractive to weevils. The strong EAG response of weevil antennae to palm wine alcohol extracts is accounted for by the volatile attractant (2*R*,5*S*)‐theaspirane. The results suggest that palm wine alcohol extracts of senesced banana leaf material could be used to lure adult *C. sordidus* to traps in the field, as part of a new, low cost weevil management technology that is affordable for smallholder banana/plantain farmers.
